# The Protective Effects of Three Polysaccharides From *Abrus cantoniensis* Against Cyclophosphamide-Induced Immunosuppression and Oxidative Damage

**DOI:** 10.3389/fvets.2022.870042

**Published:** 2022-05-02

**Authors:** Dongshuai Qu, Hongjie Hu, Shuaitao Lian, Wenjing Sun, Hongbin Si

**Affiliations:** ^1^State Key Laboratory for Conservation and Utilization of Subtropical Agro-bioresources, College of Animal Science and Technology, Guangxi University, Nanning, China; ^2^Guangxi Key Laboratory of Agricultural Resources Chemistry and Biotechnology, College of Biology & Pharmacy, Yulin Normal University, Yulin, China

**Keywords:** immunoregulation, antioxidation, natural polysaccharides, cyclophosphamide-induced immunosuppression (CTX-induced immunosuppression), *Abrus cantoniensis*

## Abstract

This study was designed to systematically elucidate the immunomodulatory and antioxidant effects of three polysaccharide fractions (ACP_60_, ACP_80_, and ACP_t2_) from *Abrus cantoniensis* on cyclophosphamide (CTX)-induced immunosuppressive mice. The experimental mice were divided into 12 groups, then modeled and administrated with different doses of three polysaccharides (50, 150, 300 mg/kg/day) by gavage. The results showed that ACP could markedly recover the CTX-induced decline in immune organ and hemocytes indexes and promote proliferation of splenocytes, earlap swelling rate, secretion of cytokines (TNF-α, IFN-γ, IL-1β, IL-6), and immunoglobulin (Ig-M and Ig-G). Additionally, ACP improved the enzymatic activities of T-SOD and GSH-PX greatly, while the level of MDA was significantly decreased in the liver. In particular, ACP_t2_ had higher immunomodulatory and antioxidant activities than ACP_60_ and ACP_80_. Based on the present findings, ACP could be utilized as an efficacious candidate for immunomodulators and antioxidants, which provide a new application prospect in the food and pharmaceutical industries.

## Introduction

*Abrus cantoniensis*, which belongs to the *Abrus* genus in the Leguminosae family, is mainly distributed in southern China, particularly in Guangdong, Guangxi, and Hunan provinces (1). This native plant has been commonly used as folk herbal medicine for many types of diseases or as a functional food for healthcare for centuries. It is reported that *A. cantoniensis* is usually used in health care food, soups, herbal tea, or beverages, such as tortoise jelly (Gui Ling-Gao), the red-canned tea “Wong Lo Kat,” and some Chinese patent medicines, such as the well-known “Jigucao capsules.” Extensive studies have indicated that *A. cantoniensis* possesses the protection against many physical illnesses such as jaundice, hepatitis, cirrhosis with ascites (2), gastric ulcer (3), sore throat (4), rheumatism, blood stasis, and internal injuries (5), and treatment of wound *in vivo* (6).

It is well documented that the effective ingredient of *A. cantoniensis* contains polysaccharides, flavonoids, phenolic compounds, alkaloids, terpenoids, saponins, and sapogenols (7). Polysaccharides, which are ubiquitous macromolecules in nature, have attracted great attention owing to their anti-oxidant (8), anti-tumor, immunomodulatory activity (9), and hypoglycemic and hypolipidemic (10) with non-toxicity and unnoticeable side effects. The immunomodulation and antioxidant properties are the most attractive bioactivities of polysaccharides. Polysaccharides derived from *Cordyceps militaris* could improve immunity and antioxidant activities (11). Some researchers found that *Desmodesmus armatus* polysaccharides can improve the antioxidant capacity in a variety of ways (12). Therefore, it is meaningful to explore the immunomodulatory effects and antioxidant activities of *A. cantoniensis* polysaccharide (ACP).

The immune system is responsible for recognizing and clearing antigenic foreign bodies through specific and non-specific immunity under normal physiological conditions, which aim to maintain an immune homeostasis balance. However, this balance can be affected and destroyed by numerous factors, such as malnutrition, infectious disease, and stressors. Furthermore, the immune imbalance will further lead to a variety of diseases (13, 14). Cyclophosphamide (CTX), a potent immunosuppressant in clinical chemotherapy, can cause severe side effects, such as damaging immune organs, avoiding the production of cytokines and antibodies, and inhibiting the proliferation of T and B lymphocytes (15). Therefore, CTX is often used as a drug to establish immunocompromised mouse models (16–18).

In our previous study, ACP has been proved to have favorable immunomodulation and antioxidation *in vitro*. However, these capacities and the key fraction of ACP in target animals have not been confirmed until now. The purpose of this investigation is to explore the immunomodulatory effect and antioxidant activity of different fractions of ACP *in vivo*. In the present study, we isolated and purified three polysaccharide fractions from *A. cantoniensis* and investigated their physicochemical features. The mice were immunosuppressed intraperitoneal injection of CTX, and then the immune indexes and antioxidant enzyme activity were evaluated and compared by gavage administration of different concentrations of three polysaccharide fractions.

## Materials and Methods

### Plant Materials and Chemicals

*Abrus cantoniensis* was purchased from Guangxi Dahong Pharmaceutical Co., Ltd. (Hechi, Guangxi, China), and then was crushed by a disintegrator (800Y, Yongkang Boou Hardware Products Co., Ltd.).

Cyclophosphamide was obtained from the Shanghai Beyotime Biotechnology Co., Ltd. dinitrofluorobenzene (DNFB) was obtained by Macklin (Shanghai, China). The ELISA kits of mouse tumor necrosis factor (TNF-α), interferon (INF-γ), interleukin (IL-6, IL-1β), Immunoglobulin M (Ig-M), Immunoglobulin G (Ig-G), and the assay kits for malondialdehyde (MDA), total superoxide dismutase (T-SOD), and glutathione peroxidase (GSH-Px) were acquired from Jiangsu Jingmei Biotechnology Co., Ltd (Jiangsu, China). The cell culture medium RPMI1640, Astragalus polysaccharides (APS), and MTT were supplied from Solarbio (Beijing, China).

### Extraction and Purification of Polysaccharides

Briefly, *A. cantoniensis* was pretreated with 95% ethanol to remove pigment for reserve use. The crude polysaccharides of *A. cantoniensis* were isolated by the water–ethanol extraction method. After the proteins were removed by the Sevag method, anhydrous ethanol was added to the extracts to make the concentration of ethanol in the system reach 80% with light stirring. After alcohol precipitation at 4°C overnight, the resulting precipitate was redissolved in ultra-pure water, and then further purified by DEAE-52 anion exchange chromatography (2.6 × 50 cm^2^). The resulting solution was eluted with ultra–pure water and 0.2 M NaCl into two peaks, the eluents were concentrated and dialyzed (cut-off MW3,500 Da) against distilled water for 36 h and vacuum-dried under freezing to obtain the refined ACP_t0_ and ACP_t2_ respectively.

In addition, the concentrated extract was added with anhydrous ethanol, and the concentration of ethanol in the system reach 40%. After centrifugation, the precipitation was collected and freeze-dried, namely, ACP_40_. According to the above operation, ethanol was added to make the final concentration of ethanol in the system reach 60 and 80% in turn; ACP_60_ and ACP_80_ were obtained, respectively. Previous *in vitro* experiments showed that ACP_60_, ACP_80_, and ACP_t2_ have excellent effects on antioxidant activity and immunomodulatory effects. Therefore, these three polysaccharide fractions were selected for further study.

### General Methods

The total carbohydrate content was determined by the phenol–H_2_SO_4_ method using glucose as a standard (19). The total uronic acid content was determined by the carbazole sulfate method using glucuronic acid as a standard (20). Protein content was measured by the Coomassie brilliant blue method using bovine serum albumin as a standard. Visible absorbance was recorded with a UV–Vis spectrophotometer (Model SP-752, China).

The ultraviolet absorbance of the ACP aqueous solution (1 mg/ml) was scanned by a U-6000PC spectrophotometer (Yuanxi, Shanghai, China) in the range of 200–600 nm. Fourier-Transform Infrared Spectroscopy (FTIR) analysis, ACP (2 mg) was ground with KBr and obtained in the range of 400–4,000/cm on a Thermo Nicolet iS5 FTIR (ThermoFisher, USA).

The molecular weight (MW) distributions of the polysaccharide samples were determined using High-Performance Gel Permeation Chromatography (HPGPC) on a TSK-GEL GMPWXL column (7.5 mm × 300 mm, 10 μm; Tosoh, Tokyo, Japan), and the monosaccharide compositions of the polysaccharide samples were analyzed by High-Performance Liquid Chromatography (HPLC) using a Thermo C18 column (250 × 4.6 mm) with 1-phenyl-3-methyl-5-pyrazolone (PMP) pre-column derivatization. The HPGPC and HPLC were carried out by using a Dionex Ultimate 3000 Liquid chromatography (ThermoFisher, USA) and a 1260 Liquid chromatograph (Agilent, California, USA).

### Animals and Experimental Design

Specific-pathogen-free (SPF) Female Kunming mice (20–22 g) were provided by Changsha Tianqin Biotechnology Co., Ltd., China (certificate number: SCXK(Xiang)2019–0014, Hunan). Before and during experiments, all of the animals were housed in pathogen-free cages with wood shavings in a room (temperature 25 ± 2°C, relative humidity 65 ± 5%) with a 12 h/12 h light–dark cycle. The mice were fed with distilled water and rodent chow *ad libitum*. All animal experimental procedures were approved by the Animal Experimental Ethics Committee of Guangxi University.

**Figure 1:**
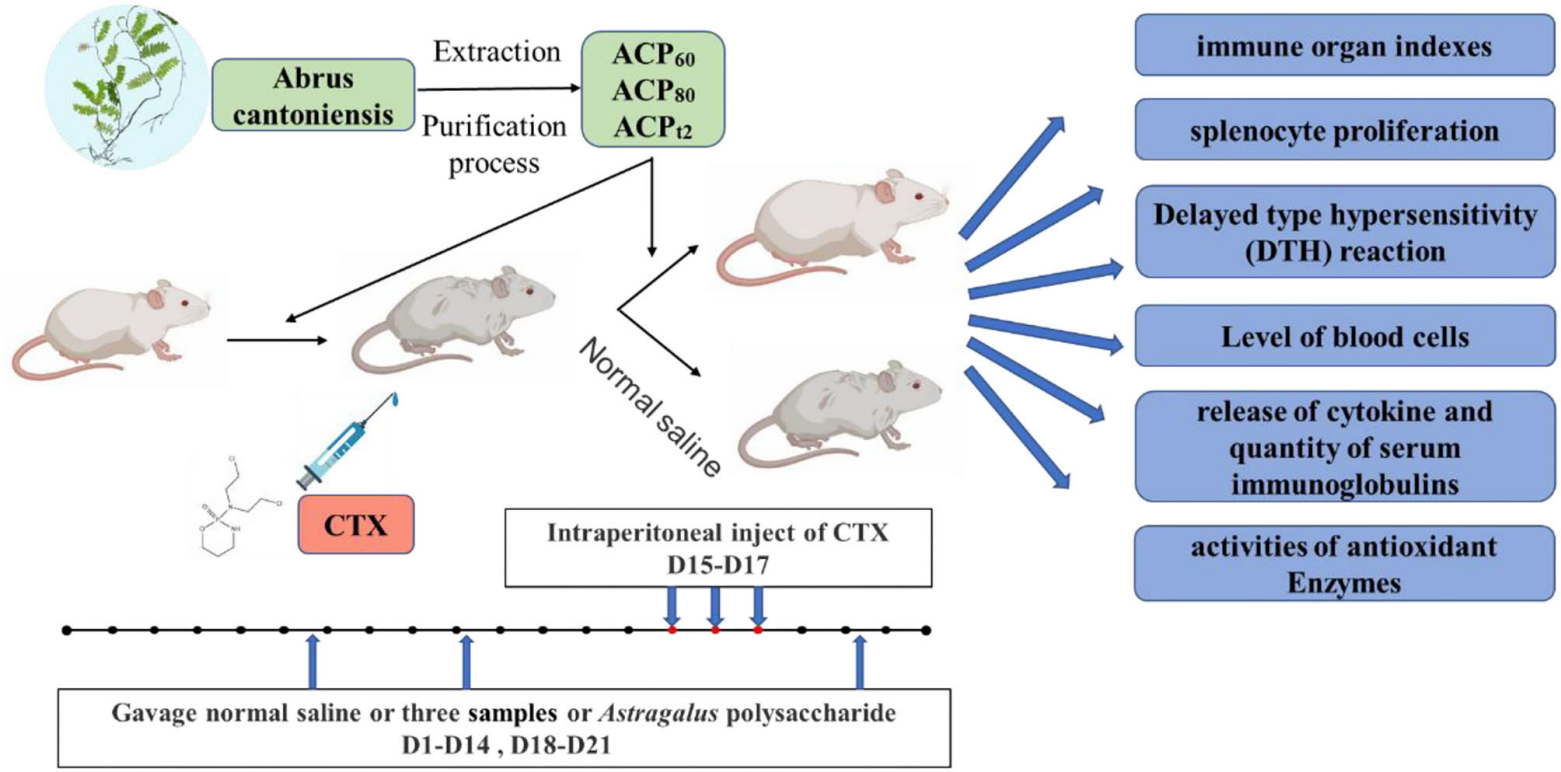
Schematic diagram of the experimental design. ACP, *Abrus cantoniensis* polysaccharides; CTX, cyclophosphamide.

After 1 week of acclimatization, the mice were divided into 12 groups randomly (each group *n* = 10): normal group, model group, positive group, ACP_60_ low-dose group, ACP_60_ medium-dose group, ACP_60_ high-dose group, ACP_80_ low-dose group, ACP_80_ medium-dose group, ACP_80_ high-dose group, ACP_t2_ low-dose group, ACP_t2_ medium-dose group, and ACP_t2_ high-dose group. The positive group was fed APS (150 mg/kg/day), and the normal group and model group were administrated 0.2 ml saline (0.1 ml/10 g) *via* gastric gavage once daily. 50/150/300 mg ACP_60_/kg/day per mouse for ACP_60_-L, ACP_60_-M, ACP_60_-H groups; 50/150/300 mg ACP_80_/kg/day per mouse for ACP_80_-L, ACP_80_-M, ACP_80_-H groups; 50/150/300 mg ACP_t2_/kg/day per mouse for ACP_t2_-L, ACP_t2_-M, ACP_t2_-H groups. The experimental procedure was implemented for 21 consecutive days. From Day 15 to Day 17, except for the normal group, all of the mice were intraperitoneally injected with 80 mg/kg/day CTX to form the immunosuppressive model. Twelve hours after the last administration, all the mice were weighed and then sacrificed by cervical dislocation to obtain blood and organs. The experimental design is outlined in Figure 1.

### Analysis of Immune Organ Indexes

After experimental mice were sacrificed by cervical dislocation, the spleen and thymus were isolated surgically under sterile conditions and weighed immediately. The immune organ indexes were calculated according to the following formula:

**The immune organ indexes (mg/g)** **=** **weight of spleen or thymus (mg)/body weight (g)**.

### Delayed-Type Hypersensitivity Reaction

On Day 16 of the experimental procedure, barium sulfide was used to shave belly fur off of each mouse with a range of about 2.0 cm × 2.0 cm, and then they were painted evenly with 50 μl 1% DNFB for sensitization. Five days later, 10 μl 1% DNFB was evenly smeared on both sides of the right earlap of each mouse. Approximately 24 h after the attack, they were executed. The ear slices (8 mm diameter) of both earlaps of each mouse were removed by puncher and weighed. The degree of DTH is reflected by the weight differentials between the right and left ear slices of each mouse.

### Assay of Splenocyte Proliferation

The splenic lymphocytes were obtained from the mouse spleen and the MTT assay was used to determine T and B lymphocyte proliferative capabilities induced by ConA and LPS, respectively. Briefly, the mice spleens were removed from sacrificed mice under germ-free conditions, and then chopped, ground, washed, and filtered to get a homogeneous cell suspension of splenocytes. The suspension was centrifuged at 2,500 rpm for 5 min and the precipitate was treated with an erythrocyte lysis solution. Subsequently, the splenocytes were washed two times by PBS and resuspended in RPMI 1640 medium with 10% newborn bovine serum. The cells were adjusted to the final density of 1 × 10^6^ cells/ml. Suspension of splenocytes (200 μl) was placed into a 96-well microtiter plate, with LPS (10 μg/ml) or Con A (10 μg/ml). Serum-free RPMI 1640 medium was used as the control, then cultured at 37°C with 5% CO_2_. After 44 h of incubation, 20 μl of MTT solution (5 mg/ml) was added to each well and then continued to incubate for 4 h. Then the supernatant was removed and we added 150 μl DMSO solution into each well. Finally, the optical density (OD) was recorded using a Microplate Reader at 570 nm (Thermo Fisher, USA).

### Effect of ACP on WBC, LYM, RBC, HGB, and PLT Levels

After experimental mice were executed, blood samples were collected by enucleating eyeball and 200 μl of which was placed into an anticoagulant tube. The contents of white blood cell (WBC), lymph (LYM), hemoglobin (HGB), red blood cell (RBC), and platelet (PLT) were measured by a Hematology Analyzer (URIT-5160Vet). The other blood was placed into a centrifugal tube and exerted to obtain the serum.

### Assay Release of Cytokine and Quantity of Serum Immunoglobulins

Blood samples in each group were collected from the ophthalmic venous plexus and centrifuged at 3,500 rpm for 15 min at 4°C to get the serum. Subsequently, the serum was collected to determine the levels of cytokines (TNF-α, IFN-γ, IL-1β, and IL-6) and immunoglobulins (IgG and IgM) using the ELISA kit according to the manufacturer's protocols.

### Effect of ACP on Activities of Antioxidant Enzymes

The same wet weight liver was taken in ice-cold normal saline. The liver tissue homogenate was prepared by an automatic homogenizer, and then, centrifuged at 2,500 rpm for 10 min at 4°C. The supernatant was collected to measure the levels of T-SOD, GSH-PX, and MDA with commercial kits.

## Results

### Physicochemical Features of ACP

**Figure 2:**
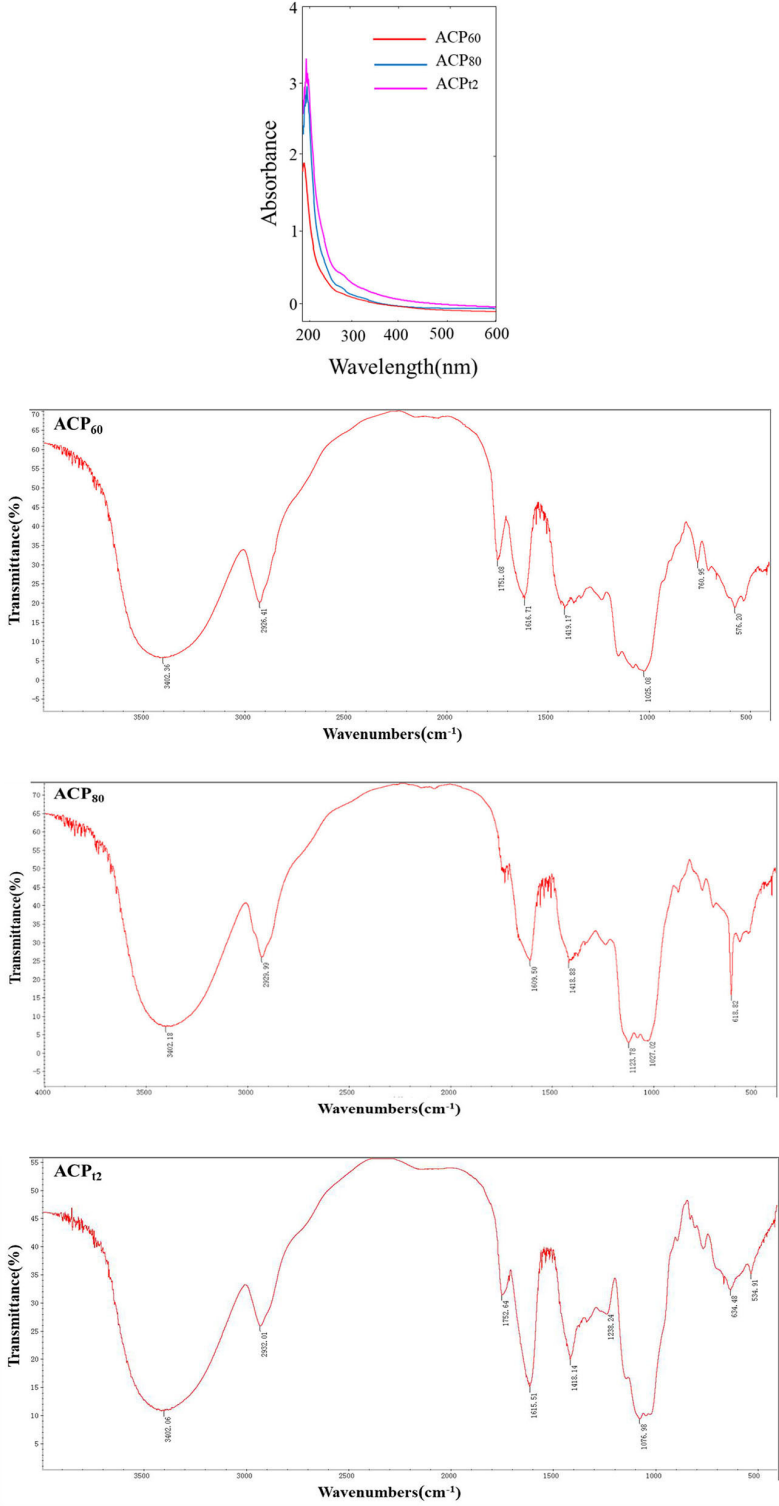
UV spectrum and FTIR spectra of ACP_60_, ACP_80_, and ACP_t2_.

ACP was obtained through water extraction, ethanol precipitation, deproteination, DEAE–cellulose anion exchange chromatography, dialysis, and lyophilization. These results revealed that the total sugar content of ACP_t2_ (99.6%) was higher than ACP_60_ (89.8%) or ACP_80_ (86.1%). Due to removing protein by the Sevag method and DEAE–cellulose anion exchange chromatography, the protein content of ACP_60_, ACP_80_, and ACP_t2_ were only 0.05%, 0.16%, and 0.11%, respectively (Table 1), which was consistent with its absorption at 280 nm in the UV spectrum (Figure 2). Additionally, there was no peak at 260 nm of UV spectra, signifying the absence of nucleic acid.

**Table 1:** The total sugar, uronic acid, and protein contents, and monosaccharide composition molecular weight of the ACP.

**Fraction**	**Total**	**Uronic**	**Protein**	**MW**	**Monosaccharide composition (%)**								
	**sugar (%)**	**acid (%)**	**(%)**	**(kDa)**	**Man%**	**GlcN%**	**Rha%**	**GlcUA%**	**GalUA%**	**Glc%**	**Gal%**	**Xyl%**	**Ara%**
ACP_60_	89.8	38.4	0.05	86.9	3.4	–	–	1.2	8.0	71.5	9.6	2.0	3.8
ACP_80_	86.1	15.5	0.16	26.2	5.7	1.0	1.7	1.7	4.9	53.5	14.3	0.2	16.8
ACP_t2_	99.6	27.9	0.11	145.6/8.9	8.8	–	1.7	3.9	22.2	11.0	25.6	6.3	16.6

The Carbazole sulfate method indicated that the uronic acid contents of ACP_60_, ACP_80_, and ACP_t2_ were 27.9%, 38.4%, and 15.5%, respectively (Table 1). The monosaccharide composition analysis indicated that ACP_t2_ was mainly composed of galactose (25.6%), galactose uronic acid (22.2%), arabinose (16.6%), glucose (11.0%), and a small amount of mannose, xylose, galactose uronic acid, and rhamnose. ACP_80_ mainly contains glucose (53.5%), arabinose (16.8%), and galactose (14.3%), while ACP_60_ mainly consisted of glucose (71.5%), galactose (9.6%), and galactose uronic acid (8.0%; Table 1).

High-performance gel permeation chromatography analysis showed that ACP_t2_ exhibited two main elution peaks. Based on known standard polysaccharides, the MW of two peaks was calculated at 145.6 and 8.9 kDa. ACP_60_ and ACP_80_ both exhibited one main elution peak: the molecular weight was estimated at MW 86.9 and 26.2 kDa, respectively.

Fourier-Transform Infrared Spectroscopy spectra of the three polysaccharides fractions are shown in Figure 2. The results indicated that the characteristic peak was at 3,400, 2,930, and 1,420/cm, which is caused by O–H, C–H, and C–O stretching vibrations, respectively (21). These characteristic absorption peaks are often used to identify polysaccharide samples. Additionally, 1,750 and 1,610/cm were probably caused by the C=O or bound water stretching vibration (22). The prominent peaks in the range of 1,000 to 1,200/cm (1,123.78, 1,027.02, and 1,025.08/cm) corresponded to C-H variable angle telescopic vibration, indicating the existence of a pyranose-ring structure in ACP_60_ and ACP_80_ (23). The band at 1,238/cm might represent the O=S=O deformation vibration, indicating that sulfates were present in the ACP_t2_. The absorption peak of 1,076.98/cm might represent C–O–H or C–O–C stretching vibrations. The modifications of characteristic groups will lead to changes in the physical and chemical properties of the polysaccharide, thus causing different biological activities.

### Effect of ACP on Immune Organs in Immunosuppressed Mice

**Figure 3:**
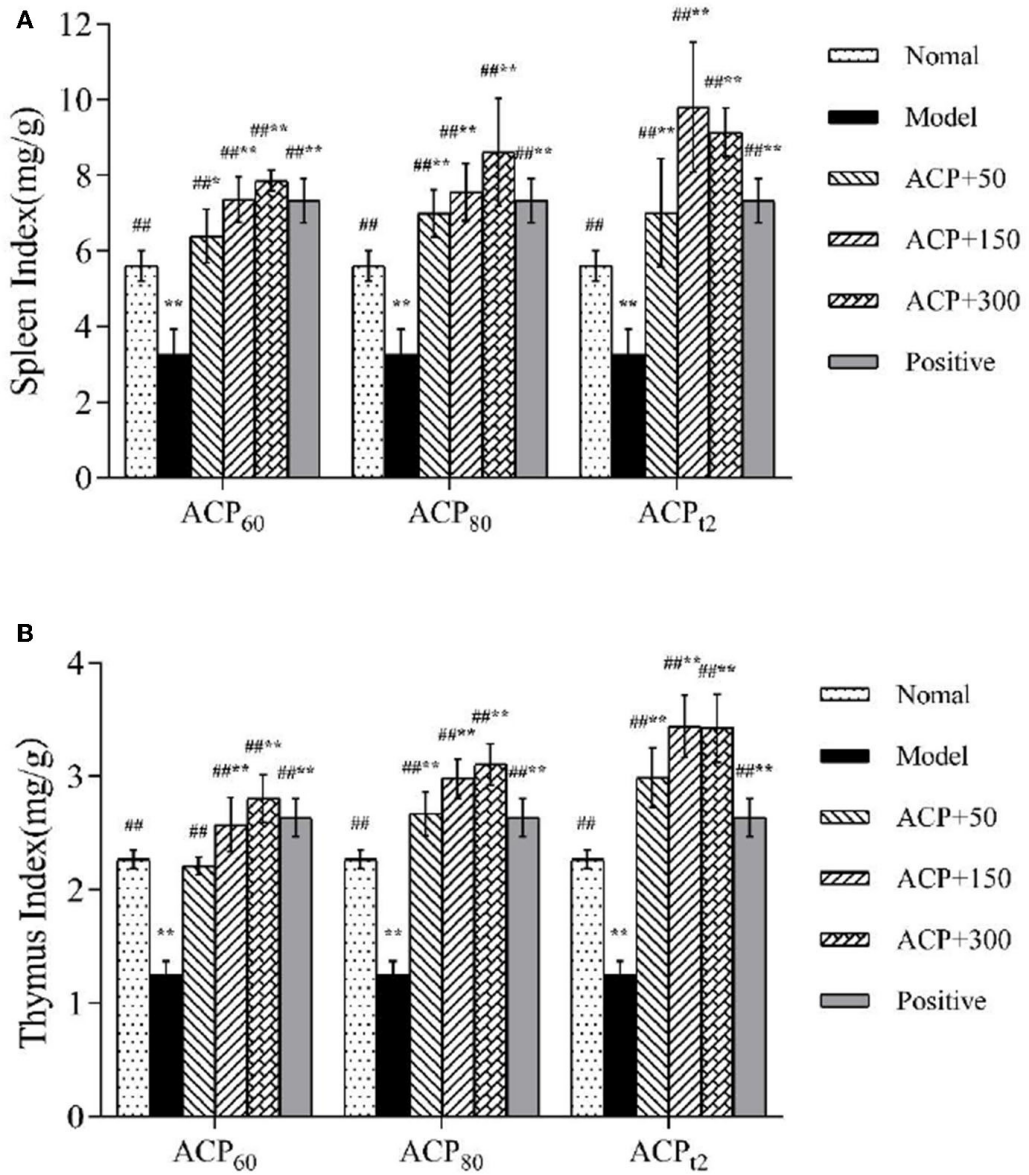
**(A)** Effect of ACP_60_, ACP_80_, and ACP_t2_ on spleen index in CTX-treated mice. **(B)** Effect of ACP_60_, ACP_80_, and ACP_t2_ on thymus index in CTX-treated mice. Data are expressed as mean ± SD. ^*^P < 0.05, ^**^P < 0.01 vs. the normal group. ^#^*P* < 0.05, ^##^*P* < 0.01 vs. the model group.

As shown in Figures 3A,B, the mice experiment was designed to examine the effect of CTX on immune organs and improvements mediated by three polysaccharide fractions. The thymus and spleen are the main immune organs in the body, thus immune function and prognosis of an organism could be reflected by the indexes of immune organs modestly (24, 25). The results showed that compared with the normal group, the indexes of the spleen and thymus in the model group radically declined (*P* < 0.01), indicating that the immunosuppressive model was established preliminarily. Supplementation with different doses of polysaccharides, the spleen and thymus indexes rose in all three polysaccharides-treated mice in varying degrees compared with the model group. Especially in the ACP_t2_ group, the dose of 150 mg/kg resulted in the most significant effect. These results demonstrated that ACP_60_, ACP_80_, and ACP_t2_ could improve the damage to immune organs of mice induced by CTX and enhance immunity. ACP_t2_ was associated with the strongest immunomodulatory effect.

### Delayed-Type Hypersensitivity Reaction

Compared with the normal group, the earlap swelling rate of model mice was significantly decreased (*P* < 0.01; Table 2), indicating that CTX damaged the cellular immunity of mice seriously. After treatment with ACP_60_, ACP_80_, ACP_t2_, and APS (positive control), the earlap swelling rate in all the groups was increased to different degrees in a dose-dependent manner compared with the model group. There was no remarkable difference in the swelling rate between the ACP_80_-H ACP_t2_-M group, ACP_t2_-H group, and normal group (*P* > 0.05). ACP_t2_ at the dose of 150 mg/kg resulted in the strongest recovery function. The results showed that three polysaccharide fractions can increase the cellular immunity of mice by enhancing the degree of earlap swelling.

**Table 2:** The effect of ACP_60_, ACP_80_, and ACP_t2_ on DTH in immunosuppressed mice.

**Groups**	**Dosage (mg/kg)**	**Earlap swelling rate (%)**
NC	/	46.36 ± 5.69^##^
MC	/	^**^21.05 ± 3.47
ACP_60_-L	50	^**^24.09 ± 4.32
ACP_60_-M	150	^**^26.69 ± 3.83
ACP_60_-H	300	^*^36.53 ± 4.11^##^
ACP_80_-L	50	^**^29.77 ± 4.44^#^
ACP_80_-M	150	^*^36.75 ± 7.79^##^
ACP_80_-H	300	40.23 ± 4.90^##^
ACP_t2_-L	50	*34.52 ± 5.56^##^
ACP_t2_-M	150	46.79 ± 5.50^##^
ACP_t2_-H	300	45.59 ± 1.90^##^
PC	150	39.94 ± 2.42^##^

*Data are expressed as mean ± SD*.

* *P < 0.05*,

** *P < 0.01 vs. the normal group*.

# *P < 0.05*,

## *P < 0.01 vs. the model group*.

### Effect of ACP on Splenic Lymphocyte Proliferation in Immunosuppressed Mice

**Figure 4:**
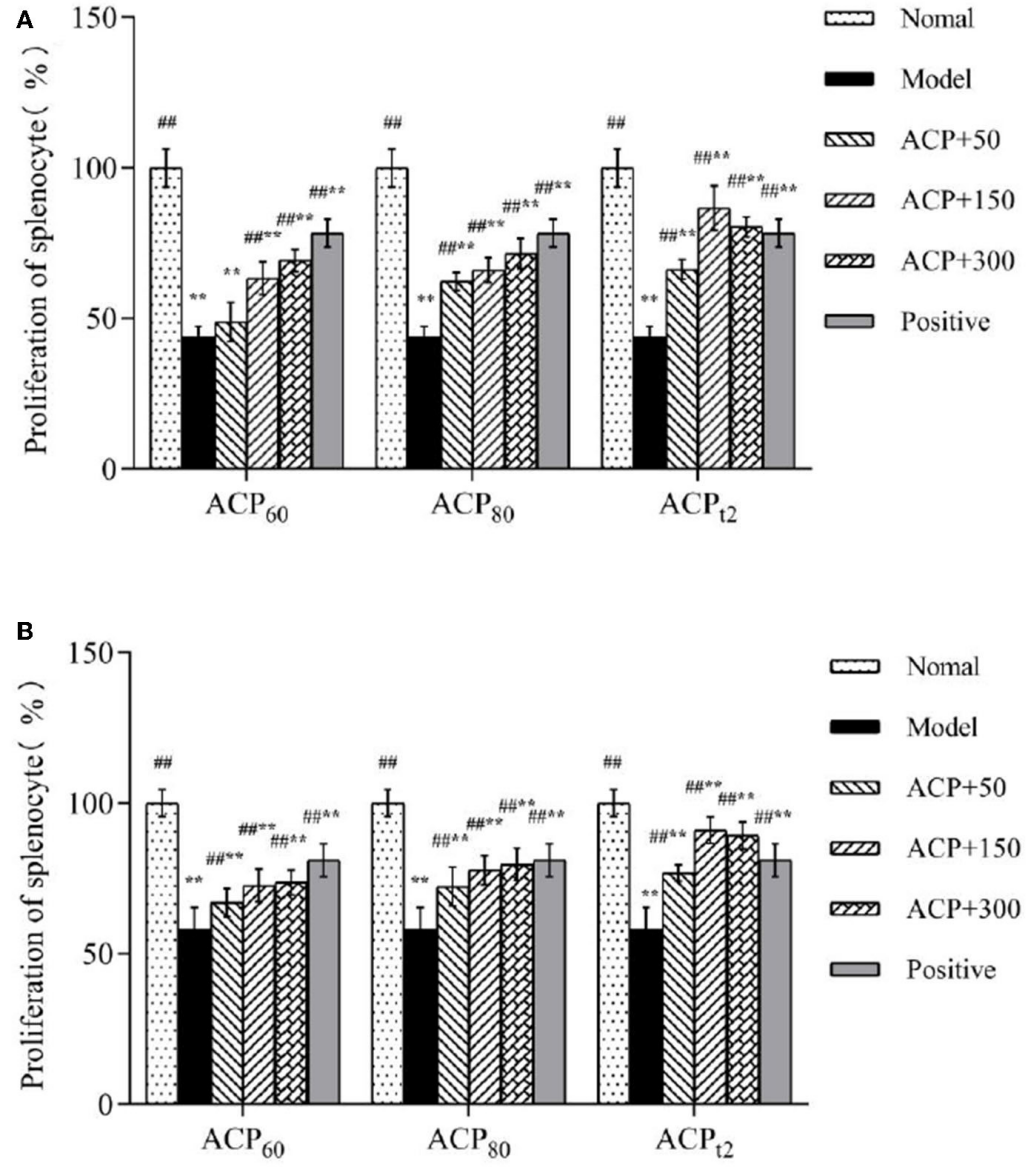
**(A)** Effects of ACP_60_, ACP_80_, and ACP_t2_ on the proliferation of LPS-stimulated splenic splenocyte in CTX-treated mice. **(B)** Effects of ACP_60_, ACP_80_, and ACP_t2_ on the proliferation of ConA-stimulated splenic splenocyte in CTX-treated mice. Data are expressed as mean ± SD. *P < 0.05, **P < 0.01 vs. the normal group. ^#^*P* < 0.05, ^##^*P* < 0.01 vs. the model group.

As shown in Figures 4A,B, compared with the normal group, the proliferation of T and B lymphocytes (43.94 and 58.20%, respectively) in the CTX-treated group declined (*P* < 0.01), which showed cellular and humoral immune functions of immunosuppressed mice were impaired. With treatment of three polysaccharide fractions, a decline in the proliferation of T and B lymphocytes was alleviated in a dose-dependent manner. Importantly, the lymphocyte proliferation rates of mice treated with the ACP_t2_ group (50, 150, 300 mg/kg) were marked higher than those of mice treated with ACP_60_ and ACP_80_ groups. The results of the proliferation assay indicated that three polysaccharide fractions had the potential for promoting the activation and proliferation of T and B lymphocytes and then enhancing the cellular and humoral immune functions of immunosuppressed mice. Similarly, positive control (150 mg/kg) showed a similar effect.

### Effect of ACP on WBC, LYM, RBC, HGB, and PLT Levels

As shown in Table 3, compared with the normal group, the levels of WBC, LYM, RBC, HGB, and PLT in peripheral blood of the model group were lower after intraperitoneal injection of CTX, which indicates that injection of CTX seriously damaged the health of mice. However, following oral administration of three polysaccharide fractions, these CTX-induced decreases were ameliorated (*P* < 0.05). Compared with the model group, the levels of ACP_80_-M, ACP_80_-H, ACP_t2_-L, ACP_t2_-M, ACP_t2_-H group, and the positive group increased significantly (*P* < 0.05, *P* < 0.01) and the effects of ACP_80_-H, ACP_t2_-M, and ACP_t2_-H groups were stronger than those of the positive group by oral administration of APS (*P* < 0.05). The effects of the same dose of ACP_60_ and ACP_80_ groups were remarkably weaker than those of the ACP_t2_ group (*P* < 0.05). It is suggested that ACP can alleviate the decrease of levels of blood cells caused by CTX, and ACP_t2_ is more effective than ACP_60_ and ACP_80_.

**Table 3:** Effect of ACP on WBC, LYM, RBC, HGB, and PLT levels.

**Group**	**WBC**	**LYM**	**RBC**	**HGB**	**PLT**
	**(10^**9**^/L)**	**(10^**9**^/L)**	**(10^**12**^/L)**	**(g/L)**	**(10^**9**^/L)**
NC	6.43 ± 0.21^##^	3.50 ± 0.20^##^	8.75 ± 0.15^##^	147.33 ± 8.74^##^	711.00 ± 15.39^##^
MC	^**^3.40 ± 0.20	^**^2.00 ± 0.26	^**^6.53 ± 0.13	^**^120.00 ± 3.61	^**^530.00 ± 15.13
ACP_60_-L	^**^4.10 ± 0.20^##^	^**^2.27 ± 0.15	^**^6.65 ± 0.24	^**^124.67 ± 4.04	^**^545.67 ± 19.50
ACP_60_-M	^**^4.77 ± 0.15^##^	^**^2.33 ± 0.15^#^	^**^7.01 ± 0.18^#^	^**^126.33 ± 5.03	^**^568.67 ± 17.79^#^
ACP_60_-H	^**^4.8 ± 0.20^##^	^**^2.53 ± 0.06^##^	^**^7.16 ± 0.13^##^	^**^133.00 ± 2.65^##^	^**^575.00 ± 8.54^#^
ACP_80_-L	^**^4.93 ± 0.15^##^	^**^2.37 ± 0.25^#^	^**^7.19 ± 0.19^##^	^**^130.33 ± 4.04^#^	^**^560.67 ± 32.19
ACP_80_-M	^**^5.40 ± 0.20^##^	^*^2.60 ± 0.10^##^	^**^7.45 ± 0.41^##^	^**^134.67 ± 5.69^##^	^**^581.67 ± 23.71^##^
ACP_80_-H	^**^5.90 ± 0.10^##^	^**^2.90 ± 0.20^##^	^**^7.80 ± 0.17^##^	^*^139.33 ± 5.13^##^	^**^637.67 ± 20.60^##^
ACP_t2_-L	^**^5.50 ± 0.10^##^	^**^2.53 ± 0.15^##^	^**^7.47 ± 0.13^##^	^**^134.003 ± 4.00^##^	^**^606.67 ± 18.18^##^
ACP_t2_-M	^*^6.03 ± 0.15^##^	3.30 ± 0.10^##^	8.41 ± 0.18^##^	^*^149.00 ± 3.00^##^	694.33 ± 14.05^##^
ACP_t2_-H	6.33 ± 0.21^##^	^*^3.13 ± 0.21^##^	^**^8.22 ± 0.28^##^	140.33 ± 4.16^##^	^*^671.00 ± 13.75^##^
PC	^**^5.78 ± 0.31^##^	^**^2.77 ± 0.12^##^	^**^7.72 ± 0.14^##^	^**^130.00 ± 4.00^#^	^**^631.67 ± 7.64^##^

*Data are expressed as mean ± SD*.

* *P < 0.05*,

** *P < 0.01 vs. the normal group*.

# *P < 0.05*,

## *P < 0.01 vs. the model group*.

### Effect of ACP on Serum Levels of Cytokines and Immunoproteins in Immunosuppressed Mice

**Figure 5:**
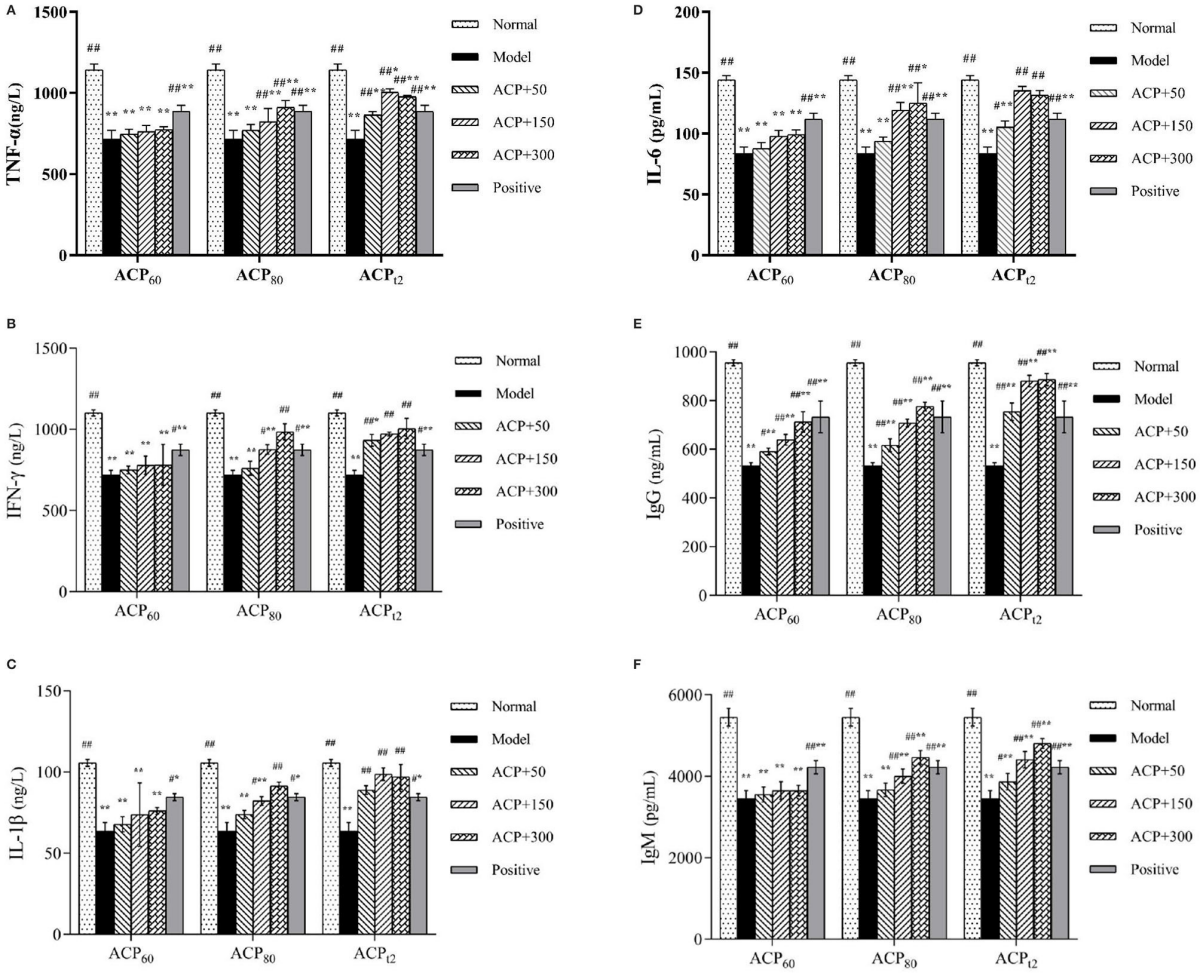
Effect of ACP_60_, ACP_80_ and ACP_t2_ on TNF-α **(A)**, INF-γ **(B)**, IL-1β **(C)**, IL-6 **(D)**, IgG **(E)** and IgM **(F)** levels in CTX-treated mice. Data are expressed as mean ± SD. ^*^P < 0.05, ^**^P < 0.01 vs. the normal group. ^#^*P* < 0.05, ^##^*P* < 0.01 vs. the model group.

The changes in cytokine concentrations in serum are shown in Figures 5A–D. After CTX injection, the concentrations of TNF-α, IFN-γ, IL-1β, and IL-6 in serum were significantly decreased (*P* < 0.01). After administration of three polysaccharides, the levels of TNF-α and INF-γ increased in a dose-dependent manner, and the concentrations of TNF-α and IFN-γ in the ACP_t2_-M group and ACP_t2_-H group were not significantly different compared with the normal group (*P* > 0.05). The concentrations of IL-1β and IL-6 also showed similar results, in which the effect of the medium dose of ACP_t2_ was stronger than that of the high dose of ACP _t2_. It may be due to the overburden of a high dose of ACP_t2_ on mice, resulting in mild toxicity. The recovery effect of ACP_t2_ groups on cytokines was stronger than that of ACP_60_ and ACP_80_ groups, which indicated that ACP_t2_ had a significant enhancing effect on the immune activity of immunosuppressed mice.

As shown in Figures 5E,F the serum IgG and IgM concentrations from the model group were lower than those of the normal group as expected (*P* < 0.01). Compared with the model group, the contents of IgG of the three polysaccharides fractions were significantly higher (*P* < 0.05, *P* < 0.01), while the content of IgM of ACP_80_-M, ACP_80_-H, and all ACP_t2_ groups significantly increased (*P* < 0.05). Based on current results, it was implied that ACP_80_ and ACP_t2_ can directly affect the strength of the immune function.

### Effect of ACP on Antioxidant Activities in Immunosuppressed Mice

**Figure 6:**
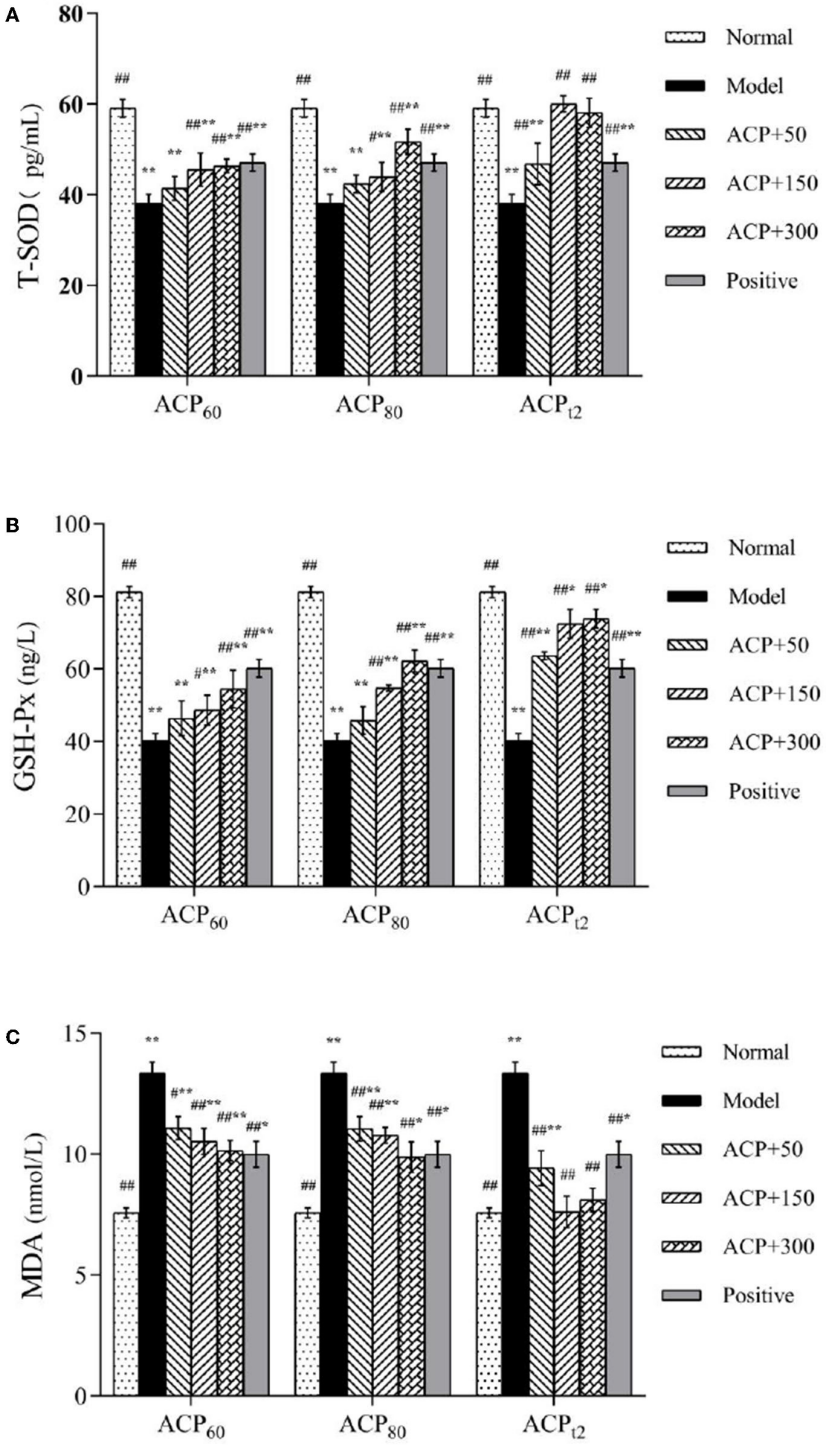
Effect of ACP_60_, ACP_80_, and ACP_t2_ on liver T-SOD **(A)**, GSH-Px **(B)**, and MDA **(C)** levels in CTX-mice. Data are expressed as mean ± SD. ^*^P < 0.05, ^**^P < 0.01 vs. the normal group. ^#^*P* < 0.05, ^##^*P* < 0.01 vs. the model group.

As shown in Figure 6, the levels of T-SOD and GSH-PX in the model group declined significantly compared with the normal group (*P* < 0.01), while the concentration of MDA in the liver of model mice presented a significant increase to 13.36 ± 0.45 nmol/L (*P* < 0.01). Compared with the model group, the activities of T-SOD and GSH-PX in all three polysaccharide groups and the positive group were increased to different degrees. The activities of T-SOD and GSH-PX in the ACP_t2_-M group and ACP_t2_-H group were significantly stronger than that of the positive control group (*P* < 0.05). At the same dose, the recovery of T-SOD and GSH-Px activities in ACP_t2_ groups was significantly stronger than that in ACP_60_ and ACP_80_ groups (*P* < 0.05). The content of MDA in the liver decreased with the increase of the concentration of sample polysaccharides. Compared with the ACP_t2_-M group, the decreasing trend of MDA content in the ACP_t2_-H group stopped, and it was speculated that ACP_t2_ had an inhibitory effect due to excessive dose. These results indicated that injection of CTX seriously destroyed the antioxidant ability of the experimental mice. However, three polysaccharides can increase the activities of T-SOD and GSH-Px and decrease the content of MDA in immunosuppressive mice. In particular, ACP_t2_ was associated with the largest effect on antioxidant ability.

## Discussion

Many factors (nutrition, disease, environment, and others) leave the body in a immunosuppressed state, which increases susceptibility to secondary infections and reduces the effectiveness of vaccines and drugs (26). However, the cellular and humoral immune mechanisms involved in the body's immune system are very complex. Host protection from immune damage and oxidative damage in the immunosuppression state is a problem worthy of attention. Immunomodulators play an important role in immune function, many herbal-based immunomodulators (such as polysaccharides, flavonoids) can enhance the host defense response, which is an effective way to improve the resistance of animals to various adverse factors (27–29). For hundreds of years, the *A. cantoniensis* has attracted wide attention for its range of biological activities. Meanwhile, the protective effect of ACP on host immunosuppression and oxidative damage has not been reported. In this study, CTX was used to establish a mouse immunosuppression model to study the regulation of ACP on immune function and the protective effect of oxidative damage in mice.

As the main immune organs, the thymus and spleen can directly affect the level of immunity which are the locations where immune cells grow and proliferate (13). Studies have shown that polysaccharides from *Mulberry leaf* can restore the immune organ atrophy induced by CTX, thus promoting the development of the immune system (30). In this study, after dealing with CTX by intraperitoneal injection, the spleen and thymus indexes of mice came down markedly, indicating that the immunosuppressive model was established successfully. All three natural polysaccharides increased these indexes, suggesting that polysaccharides can protect the body's immune organs and thus improve the host's immune function.

Lymphocytes are the core cells in the immune system. It plays a crucial role in humoral immunity and cellular immunity, mainly undertaking the task of the acquired immune response. Lymphocytes can be activated after the process of antigen presentation and mitogen stimulation (31). T lymphocyte is the main component of cellular immunity, which could directly inhibit and destruct target antigens, such as tumor cells and viruses. In humoral immunity, the corresponding antigen could activate B lymphocyte, which is differentiated into plasma cells to produce a variety of antibodies (32). The proliferation process of T lymphocyte was induced by ConA (T-cell mitogen) and B lymphocyte was induced by LPS (B-cell mitogen), which aims to evaluate the functions of cellular immunity and humoral immunity. Previous studies have shown that natural polysaccharides can significantly improve splenic lymphocyte proliferation in immunosuppressed mice induced by CTX. Zhou et al. obtained a polysaccharide from *Lonicera japonica* (LJP) and found that LJP (60,120 mg/kg) could significantly improve the proliferation of spleen lymphocytes in immunosuppressed mice induced by CTX (14). Li et al. (33) found that different doses of GFP-2 combined with ConA or LPS significantly increased the proliferation of T and B lymphocytes, and there was a significant dose correlation. This suggests that polysaccharides protect the body's immune cells. Our results are consistent with the above studies, suggesting that ACP enhances host immune function by stimulating lymphocytes. In particular, the effect of ACP_t2_ on lymphocyte proliferation is better than that of ACP_60_ and ACP_80_.

DTH is also known as type IV hypersensitivity (34). It is a cytoimmunity mediated by T lymphocytes, which encompasses two phases: in the sensitization phase, when the body is first exposed to an antigen, CD4^+^ T cells recognize the antigen presented by APC and are activated (35). DNFB diluent was able to combine with abdominal wall skin protein to form a complete antigen, which stimulates T lymphocytes to proliferate into sensitized lymphocytes. After 5–7 days, the earlap of the mice was smeared using the diluent of DNFB. Next, in the effector phase, when the activated T cells encounter the antigen again, they proliferate rapidly to become effector T cells. These effector T cells release Th1 cytokines, (IL-3, MCSF), and chemokines to recruit mononuclear macrophages, and meanwhile, IFN-γ and TNF-α induce the activation of macrophages (36, 37). Inflammation continues to amplify and accumulate, which caused regional earlap swelling. Thus, the response intensity of DTH is reflected by the degree of earlap swelling, which also indicated a level of cellular immunity. In our study, we found that the immunosuppressed mice administrated with medium and high dosages of ACP_t2_ appeared to have a higher degree of swelling compared with the control group. The above results indicated that ACP_t2_ can protect the mouse immune system effectively.

Hemocyte includes WBC, LYM, RBC, HGB, and PLT. Immune-related genes could be expressed in hemocytes. Many immunocompromised diseases or infectious diseases are closely related to the number of WBCs (25). Lymphocytes participate in cellular and humoral immunity of the body (38, 39). Red blood cells can also play a role in immune response, such as antigen presentation and cancer immunotherapy (40). Hemoglobin transports oxygen for all organs of the body and some research demonstrated that HGB can interact with the innate immune system either directly or through binding to pathogen-associated molecular patterns (41). Platelets are closely related to the inflammatory process of many types of diseases and exhibit complex interactions with their circulating cells and blood vessel walls (42). In this experiment, the level of WBC, LYM, RBC, HGB, and PLT decreased by the injection of CTX. However, those levels in mice administrated with ACP got enhanced significantly compared with the model group. This indicated that ACP could improve the decline of RBC, WBC, Hb, and PLT levels induced by CTX and restore mice immunity.

As the intercellular signaling peptides, cytokines are especially important in the immune system. In the body's immune response, it can promote the proliferation and differentiation of target cells, induce receptor expression, and enhance the body's resistance to infection, thus playing an important role in regulating the body's immune and inflammatory response. TNF-α, produced mainly by activated macrophages, plays different roles in regulating multiple developmental and immune processes, such as killing and inhibiting tumor cells (43). IFN-γ is an important immune response molecule, which can activate macrophages and produce inducible nitric oxide synthase (iNOS), to promote NO synthesis. IFN-γ is the first line of defense against antiviral infection, especially it plays a crucial role in cellular immunity (44). As a key multifunctional cytokine, IL-1β is involved in both autoimmune inflammatory responses and a range of cellular activities, such as cell proliferation, differentiation, and apoptosis (45, 46). Produced by activated monocytes, fibroblasts, endothelial cells, and so on, IL-6 can stimulate lymphocytes proliferation and promote the production of immunoglobulin by B cells (47). Previous studies have shown that some polysaccharides can activate immune cells and increase cytokines and immunoglobulin levels, thereby alleviating CTX-induced immune damage. *Dendrobium sonia* polysaccharides (DSP) can significantly increase the secretion of TNF-α, IL-6, and IFN-γ in serum, thus showing good immune-enhancing ability (48). *Millettia speciosa Champ* polysaccharides can improve CTX-induced immunosuppression by upregulating serum levels of IL-2, IL-4, IL-10, TNF-α, and IgG (49). Immunoglobulin is a kind of protein that is used to protect against a variety of pathogens, including bacteria, viruses, cancer cells, and other harmful substances, which is mainly secreted by plasmacyte (50). IgM is the immunoglobulin with the largest molecular weight, which is mainly secreted and in humoral immune response first, accounting for 5–10% of the total immunoglobulin in serum. IgM has powerful functions of sterilization, complement activation, immune conditioning, and agglutination (51). IgG is the most important antibody in the body, accounting for approximately 75% of the total immunoglobulin content in the serum. It has the functions of a neutralizing virus, antibacterial, and immune regulation, and plays an important role in neonatal anti-infection (52). The decline in the expression of TNF-α, IFN-γ, IL-1β, IL-6, Ig-M, and Ig-G in serum of the CTX model group was found in our study, which means the immune system of mice got damaged by the injection of CTX. It was observed that there was a similar trend in the production of cytokines and immunoglobulins among the groups. The results showed that ACP can promote the secretion of TNF-α, IFN-γ, IL-1β, IL-6, Ig-M, and Ig-G, regulate the immune function of the system to some extent, and alleviate immune damage.

Cyclophosphamide is an important chemotherapy drug. At the same time, it can also damage normal cells (53). It not only causes immune suppression but also affects the antioxidant capacity of the body to varying degrees (54, 55). Under normal conditions, reactive oxygen species (ROS) in organisms are controlled at a low range to form homeostasis, yet the stimulation of CTX can cause the imbalance of ROS *in vivo*. MDA is an important product of peroxidation between ROS and polyunsaturated fatty acids, which can cause cross-linking polymerization of proteins, nucleic acids, and other life macromolecules, and has cytotoxicity. MDA not only affects the activities of respiratory-related enzymes but also aggravates the peroxidation damage of biofilm (56, 57). Therefore, the level of MDA can indirectly reflect the degree of cell injury. T-SOD can catalyze the disproportionation of superoxide radicals to oxygen and hydrogen peroxide, which plays an essential role in oxidant–antioxidant homeostasis (58). GSH-Px, a vital part of peroxidase, can protect the structure and function of the cell membrane from the interference and damage of peroxides in the body (59). In previous research studies, it had been figured out that *Chimonanthus nitens Oliv* polysaccharides and *Gynostemma pentaphyllum* polysaccharides protected immunosuppressed mice by relieving oxidative damage (60, 61). The activities of GSH-PX and T-SOD in the liver of mice in the ACP group increased, suggesting that ACP has an obvious antagonistic effect on the decrease of antioxidant capacity induced by CTX. The result shows that ACP can not only alleviate the symptoms of immunosuppression but also prevent liver oxidative damage. This is consistent with the previous report.

## Conclusions

This study aimed to evaluate and compare the protective effects of three polysaccharide fractions *A. cantoniensis* on CTX-induced immunosuppression and oxidative damage in mice. The results showed that ACP_60_, ACP_80_, and ACP_t2_ had significant immuno-enhancing effects at different levels through inhibiting attenuation of spleen, thymus, and hemocytes, elevating the proliferation ability of T and B lymphocytes, improving the cellular immune function, upregulating the secretion of cytokines (TNF-α, IFN-γ, IL-1β, IL-6, IgG, and IgM). Meanwhile, ACP improved CTX-induced oxidative damage by improving the activities of T-SOD and GSH-Px and reducing the level of MDA in the liver. Among them, ACP_t2_ may be an effective contributor to the immunomodulatory and antioxidant effects of *A. cantoniensis*. These results provide a theoretical basis for the development and application of ACP as a new immunopotentiator, and can also be used as an immunostimulant in the food and pharmaceutical industry to reduce immunosuppression caused by various factors.

## Data Availability Statement

The original contributions presented in the study are included in the article/supplementary material, further inquiries can be directed to the corresponding authors.

## Ethics Statement

The animal study was reviewed and approved by Animal Experimental Ethics Committee of Guangxi University.

## Author Contributions

HS and WS initiated the project. DQ and HH were responsible for designing experimental ideas and data analysis, protocol designing, and draft editing. DQ, HH, and SL conducted the experimental work, and processed the data. HS and WS supervised and conducted the experimental work. All authors contributed to the article and approved the submitted version.

## Funding

The project was supported by Project funded by High-level Talents-Assistant Professor-Wenjing Sun Foundation (A3340051007), the National Natural Science Foundation of China (31760746), and the Key Research and Development Plan of Guangxi (AB19245037).

## Conflict of Interest

The authors declare that the research was conducted in the absence of any commercial or financial relationships that could be construed as a potential conflict of interest.

## Publisher's Note

All claims expressed in this article are solely those of the authors and do not necessarily represent those of their affiliated organizations, or those of the publisher, the editors and the reviewers. Any product that may be evaluated in this article, or claim that may be made by its manufacturer, is not guaranteed or endorsed by the publisher.
